# Clinicopathological study of centrally necrotizing carcinoma of the breast

**DOI:** 10.1186/s12885-015-1305-y

**Published:** 2015-04-14

**Authors:** Yanling Zhang, Yurong Ou, Donghong Yu, Xiang Yong, Xiaoli Wang, Bo Zhu, Qiong Zhang, Lei Zhou, Zhaogen Cai, Zenong Cheng

**Affiliations:** Department of Pathology, The First Affiliated Hospital of Bengbu Medical College, 287 Changhuai Road, Bengbu, Anhui 233000 People’s Republic of China

**Keywords:** Breast tumor, Cancer, Basal-like, Necrosis, Immunological classification, Prognosis

## Abstract

**Background:**

Centrally necrotizing carcinoma of the breast (CNC) represents a newly-identified subset of breast cancer. The clinical and pathological characteristics of this breast cancer subtype are not yet completely understood.

**Methods:**

We assessed the clinicopathological characteristics of 73 cases of CNC and 30 control cases of high-grade infiltrating ductal carcinoma (IDC) with focal necrosis based on light microscopy and immunohistochemical staining for estrogen receptor, progesterone receptor, Cerb-B2/HER2, Ki-67, epidermal growth factor receptor, cytokeratin 5/6, smooth muscle actin, S-100 protein, p63 and CD10.

**Results:**

All the tumors showed extensive central necrotic or acellular zones with different degrees of fibrotic or hyaline material surrounded by ring-like or ribbon-like residual tumour tissue which were usually high-grade IDCs. The central necrotic zone accounted for at least 30% of the cross-sectional area of the tumor. Thirty-six cases (49.3%) showed a component of ductal carcinoma in situ. The tumorous stroma around the central necrotic zone was accompanied by myxoid matrix formation in 28 cases (40%). Lymphocytic infiltration was present in 53 cases (72.6%). Granulomatous reactions were detected at the periphery of the tumors in 49 cases (67.1%). Immunohistochemistry showed greater expression of basal-like markers (72.2%, 52 cases) than myoepithelial markers (60.6%, 43 cases), both of which were significantly higher than in controls (26.7%, 8 cases) (P < 0.001). According to molecular typing, most CNCs were basal-like subtype (37 cases, 50.7%). Follow-up data were available for 28 patients. Disease progression occurred in 11 patients. The combined rate of recurrence, distant metastasis or death was significantly higher in CNC patients compared with controls (P < 0.05).

**Conclusions:**

CNC was associated with distinctive clinicopathologic features mostly characterized as basal-like type. Its high proliferative activity, highly-aggressive biological behavior, and high rates of recurrence and metastasis, suggest that CNC should be classified as a new type of breast carcinoma.

## Background

Centrally necrotizing carcinoma of the breast (CNC) represents a newly-identified subset of breast cancer, usually included in “infiltrating ductal carcinoma, not otherwise specified”. It has been described as a “fibrotic focus in invasive ductal carcinoma of the breast” [1], and as “high-grade invasive ductal carcinomas with large central acellular zones” [2,3], since its first description by Jimenez et al. in 2001 [4]. There have only been four reports of CNC to date; two case reports, and two other studies [4-7]. The clinical and pathological characteristics of this breast cancer subtype thus remain poorly understood. We therefore retrospectively analyzed the clinicopathological features, immunophenotype, and biological behavior of 73 cases of CNC definitively diagnosed in our pathology department, and conducted a literature review.

## Methods

### Materials

Seventy-three patients with CNC of the breast were initially diagnosed as infiltrating ductal carcinoma (IDC) from November 2008 to April 2014 at the Department of Pathology, First Hospital Affiliated to Bengbu Medical College. A total of 1,998 breast cancer cases were admitted to our hospital between January 2011 and December 2012, though not all had complete clinical and pathological data. During this period, 54 cases of CNC were diagnosed in our pathology department, accounting for 2.7% of all breast cancers. Thirty patients with high-grade IDCs with minimal necrosis (necrotic area <30% of the cross-sectional area of the tumor) were randomly selected as controls. The two sets of tumors were matched for tumor size, nodal status and histologic grade (P > 0.05) (Table 1). According to the diagnostic criteria of Tsuda and Jimenez et al. [2,4], CNC met the following conditions: (i) tumor composed of extensive central necrosis with varying degrees of fibrotic, hyalinized matrix or scar tissue; (ii) necrotic or acellular zones accounted for ≥30% of the cross-sectional area of the tumor (necrotic area measured under a microscope, compared with the total tumor area by microscopic or gross inspection); (iii) viable tumor tissue was mostly high-grade IDC (histological grade assessed according to the WHO-recommended Elston-Ellis Classification of Breast Carcinoma 2003 edition [8] and modified Scarff-Bloom-Richardson system [9]).

**Table 1:** **Clinicopathological features in CNC and control patients**

	Experimental group	Control group	P
	(CNC)	(IDC with localized necrosis)	
Mean tumor size (cm)	2.49	2.34	>0.05
Negative lymph nodes	40/62	19/30	0.988
IDC grade 3	67/73	27/30	0.771

IDC, infiltrating ductal carcinoma.

### Methods

Surgical specimens were fixed with 10% neutral buffered formalin and embedded in paraffin blocks. Tissue blocks were cut into 4-μm slides, deparaffinized in xylene, rehydrated with a graded alcohol series, and immunostained with the following antibodies: estrogen receptor (ER; 1D5), progesterone receptor (PR; PgR 636), HER2 (Polyclone), Ki-67 (MIB-1), epidermal growth factor receptor (EGFR; EGFR.113), cytokeratin 5/6 (CK5/6; D5/16B4), smooth muscle actin (SMA; 1A4), S-100 protein (4C4.9), p63 (4A4) and CD10 (MX002). Sections were stained using a streptavidin-peroxidase system (KIT-9720, Ultrasensitive TM S-P, MaiXin, China). The chromogen used was diaminobenzidine tetrahydrochloride substrate (DAB kit, MaiXin, China), and sections were slightly counterstained with hematoxylin, dehydrated and mounted. Positive and negative controls were included for each antibody, according to the kit instructions.

Immunopositivity for ER or PR was defined as >10% of tumor cells with nuclear immunoreactivity for ER or PR, respectively. According to the standard guide [10], >10% tumor cells with membranous staining 3+ for HER2 was considered as positive expression. More than 5% of tumor cells with cytoplasmic and/or membranous staining for CK5/6, SMA and CD10, nuclear reactivity for S-100 protein and p63, and membranous reactivity for EGFR were considered to indicate positivity for the respective antigen. Nuclear Ki-67 immunoreactivity was considered as positive expression.

Written informed consent for participation in the study was obtained from participants and accompanying images. A copy of the written consent is available for review by the Editor-in Chief of this Journal.

### Molecular typing

Tumors were divided into five types based on the immunohistochemical results and the classification of Carey et al. [11]: luminal-A (ER+ and/or PR+, HER2−), luminal-B (ER+ and⁄or PR+, HER2+), basal-like (ER−, PR− and HER2−, but positive for ≥1 basal-like markers), HER2 overexpressing (ER−, PR−, HER2+), and null phenotype (negative for all the above markers).

### Follow-up data

Follow-up data were obtained by asking patients or their relatives. Disease-free survival (DFS) was defined as the period from the first surgery for breast carcinoma to recurrence, metastasis, death, or the last follow-up. Disease progression was defined as recurrence, metastasis or death due to breast carcinoma. Overall survival (OS) was defined as the period from the first surgery to death or last follow-up.

### Statistical methods

The significance of differences between the CNC and control groups, and differences within the CNC group were analyzed by Student’s *t*-tests for means and χ^2^ tests for frequencies. Values of P < 0.05 were considered statistically significant.

This study was approved by the Ethics Committees of the First Affiliated Hospital of Bengbu Medical College and conducted in accordance with the ethical guidelines of the Declaration of Helsinki.

All the authors read and approved the final manuscript.

## Results

### Clinical information

**Figure 1:**
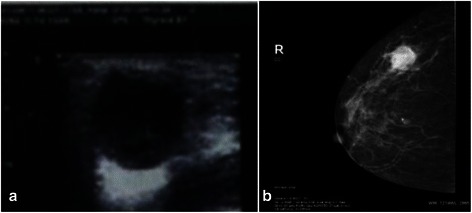
The imaging features of CNC. **(a)**: Ultrasonography indicated a well-defined, heterogeneous, hypoechoic lesion. **(b)**: The tumor was manifested as a irregular nodule with a heterogeneous, higher-density shadow, associated with tortuous vasculature around the tumor.

All 73 patients were women, with a mean age of 51.8 years (range, 34–80 years). These patients accounted for 2.7% of all breast carcinoma patients at our hospital during the same period. The clinical genetic testing or strong family history were not available. Ultrasound examinations were performed in 26 patients, of whom 14 presented with well-defined, heterogeneous, hypoechoic lesions (Figure 1a). Mammary gland molybdenum targets were available in 30 patients, six of whom showed Breast Imaging Reporting and Data System (BI-RADS) category 4 and 26 of whom showed BI-RADS 5. Well-defined, heterogeneous, higher-density shadows were present in 21 cases (Figure 1b), while blurred or spiculate borders were seen in nine cases and markedly increased vessels in 18 cases. Breast-conserving surgery was performed in one patient (without axillary lymph node dissection) and modified radical mastectomy in 62 patients, of whom 64.5% (40/62) were axillary node negative. The remaining 35.5% (22/62) had axillary lymph node metastases. The numbers of lymph node metastases were one in five patients, two in six patients, three in two patients, four in one patient and more than four in eight patients (mean, 5.8). Follow-up data were available in 28 patients, with a mean follow-up time of 21.2 months (range, 7.0–40.0 months). The mean and median DFS were 13.8 and 14.5 months, respectively. Disease progression occured in 11 patients, including one patient with regional recurrence in the ipsilateral chest wall and ten patients with distant metastases (three pulmonary, three brain, two bone, one liver, and one supraclavicular lymph nodes and mediastinum). At the time of the last follow-up, one patient had died of breast carcinoma. There was no significant relationship between disease progression of CNC and the relative size of the necrotic zone (P > 0.05). Follow-up data were available for 11 control patients, with a mean follow-up time of 31.6 months. No controls had regional recurrence, distant metastasis, or death at the last follow-up.

### Pathological features

**Figure 2:**
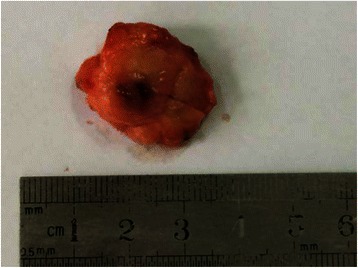
The tumors showed unicentric nodules. The cut surface had a white to tan appearance, accompanied by necrosis in the central region.

The mean tumor diameter was 2.49 cm (range, 1.0–6.5 cm). Sixty-seven patients (91.8%) had unicentric nodules (Figure 2), two had double nodules (2.7%), and four had lobulated lesions (5.5%). Most tumors had well-defined boundaries, but 14 (19.2%) with infiltrative edges. The cut surface was gray, sallow, or reddish-brown, with necrosis or cystic degeneration in the central region (Figure 2).

**Figure 3:**
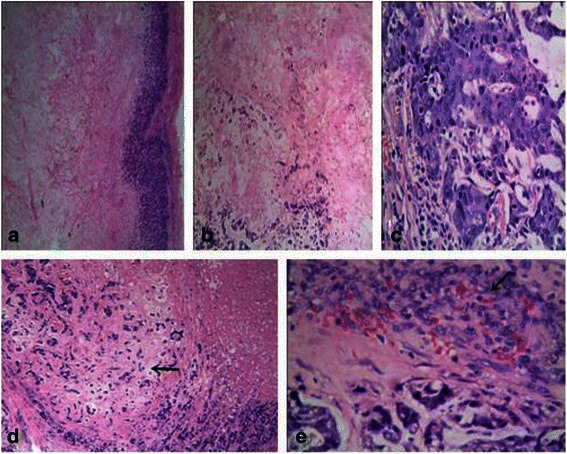
Microscopical findings of CNC. **(a)**: The tumor presented with an extensive central necrotic or acellular zone surrounded by ring-like or ribbon-like residual tumor tissue. The transition between the central necrotic or acellular zone and the viable tumor tissue was abrupt (×4). **(b)**: The central zone of the tumor showed coagulative necrosis with pink, fine granules combined with fibrotic or hyaline material and a tumorous stroma around the central necrotic zone, accompanied by myxoid matrix formation (×10). **(c)**: The tumor cells were arranged in cord-like or nest-like patterns. Most of the tumor cells showed evident atypia, prominent nucleoli, and frequent mitotic figures (×40). **(d)**: Focal cartilaginous metaplasia was present in this case (→)(×10). **(e)**: The periphery of the tumors demonstrating the granulomatous reaction(→) (×40).

Microscopically, all the tumors presented with an extensive central necrotic or acellular zones surrounded by ring-like or ribbon-like residual tumor tissue. The transition between the central necrotic or acellular zone and viable tumor tissue was usually abrupt (Figure 3a).

The central necrotic zone varied in size and morphology. The morphological features of the central necrotic or acellular zone were: (i) coagulative necrosis as the main feature in 47 cases (63.4%), shown as a pink, fine-granular area with discernible hemorrhage, necrotic tissue fragments, or ‘ghost’ cells, and scattered or focal fibrotic or hyaline material (Figure 3b); (ii) fibrotic, hyaline material or scar tissue occupied most of the central zone in 24 cases (32.9%), arranged in cords or sheets, with a small amount of necrotic cellular debris or ‘ghost’ cells; (iii) the remaining two cases (2.7%) consisted of infarction without varying degrees of fibrotic or hyaline material, in which the original outline of organizational structure could be seen indistinctly.

The residual tumor tissue surrounding the central necrotic zone varied, but most cases showed high-grade IDCs (67 cases, 91.8%). The tumor cells were arranged in cord-like or nest-like patterns, lacking tube-like structures. Most tumor cells showed evident atypia, prominent nucleoli, and frequent mitotic figures (Figure 3c). Multinucleated giant cells were present in several cases. A ductal carcinoma in situ component was present in 36 cases (49.3%); five cases (6.8%) were associated with a mucinous carcinoma component, two with invasive micropapillary carcinoma, one with intraductal papillary carcinoma. Focal metaplasia was present in six cases (8.2%), including squamous metaplasia in four, spindle cell differentiation in one, and cartilaginous metaplasia in one (Figure 3d).

The tumorous stroma around the central necrotic zone was accompanied by myxoid matrix formation in 28 cases (40%), lymphocytic infiltrates were present in 53 (72.6%), histiocytic reaction in five, and calcification in 20 cases.

The periphery of the tumor demonstrated a well-defined boundary in 59 cases (80.8%), including 49 cases (67.1%) in which the tumor was enclosed by fibers indicating a granulomatous reaction (Figure 3e). The remaining 14 cases (19.2%) showed poorly-defined boundaries with infiltrative edges. Vascular invasion was seen in ten cases (13.7%) and nerve infiltration in five. There were no significant associations between the clinicopathological features of CNC and the relative size of the necrotic zone (P > 0.05) (Table 2).

**Table 2:** **Relationship between clinicopathological features and extent of necrosis in 73 patients with CNC**

Clinicopathological feature		Case	≥30% but <50% necrosis	≥50% but <70% necrosis	≥70% necrosis	P
			(7, 9.6%)	(25, 34.3%)	(41, 56.2%)	
IDC	Grade 3	67	7 (100.0%)	23 (92.0%)	37 (82.2%)	0.685
	Grade 2	6	0 (0.0%)	2 (8.0%)	4 (9.8%)	0.685
	Grade 1	0	0 (0.0%)	0 (0.0%)	0 (0.0%)	—
Ductal carcinoma in situ		36	4 (57.1%)	13 (52.0%)	19 (46.3%)	0.823
Mucinous carcinoma		5	1 (14.3%)	3 (12.0%)	1 (2.4%)	0.235
Intraductal papillary carcinoma		1	0 (0.0%)	0 (0.0%)	1 (2.4%)	0.673
Invasive micropapillary carcinoma		2	0 (0.0%)	1 (4.0%)	1 (2.4%)	0.835
Metaplasia		6	0 (0.0%)	4 (16.0%)	2 (4.9%)	0.198
Myxoid stroma		28	1 (14.3%)	11 (44.0%)	16 (39.0%)	0.357
Lymphocytic infiltration		53	5 (71.4%)	17 (68.0%)	31 (75.6%)	0.796
Granulation tissue proliferation		49	3 (42.9%)	19 (76.0%)	27 (65.9)	0.248
Vascular invasion		10	0 (0.0%)	4 (16.0%)	6 (14.6%)	0.534
Perineuronal invasion		5	1 (14.3%)	1 (4.0%)	3 (7.3%)	0.625
Expression of myoepithelial markers		43	4 (57.1%)	19 (76.0%)	20 (48.8%)	0.092
Expression of basal-like markers		52	5 (71.4%)	22 (88.0%)	25 (61.0%)	0.063
Molecular classification	Basal-like subtype	37	3 (42.9%)	9 (36.0%)	25 (61.0%)	0.132
	Luminal A	20	3 (42.9%)	9 (36.0%)	8 (19.5%)	0.217
	Luminal B	7	1 (14.3%)	5 (20.0%)	1 (2.4%)	0.057
	HER2-overexpressing	6	0 (0.0%)	0 (0.0%)	6 (14.6%)	0.078
	Null subtype	3	0 (0.0%)	1 (4.0%)	2 (4.9%)	0.834
Disease progression		11	1 (14.3%)	2 (8.0%)	8 (19.5%)	0.796

### Immunohistochemical analysis

**Figure 4:**
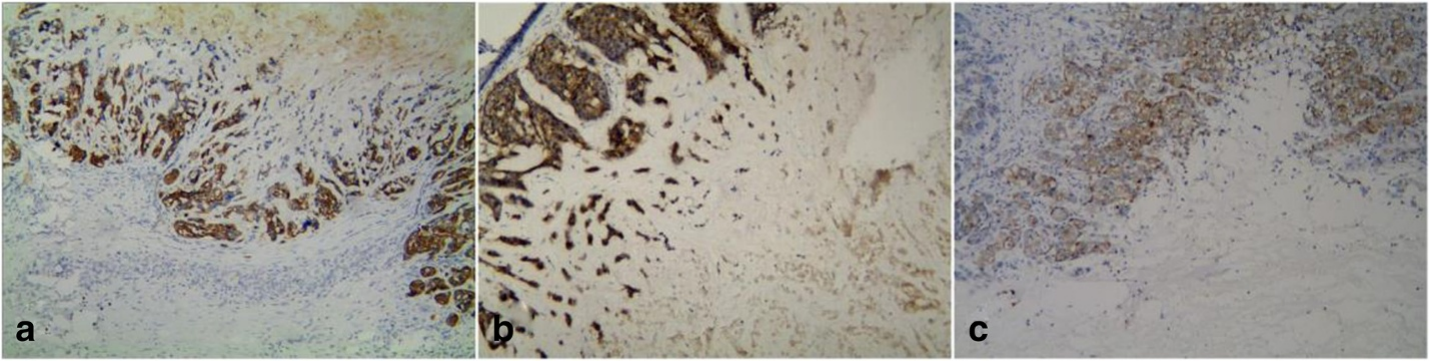
Immunohistochemical findings of CNC. **(a)**: The tumor cells were diffusely, strongly-positive for CK5/6 (×10). **(b)**: The tumor cells were diffusely, strongly-positive for EGFR (×10). **(c)**: The tumor cells were positive for CD10 (×10).

There was extensive cellular proliferation. The Ki-67-labeling index was >50% in 62 cases (84.9%), and >70% in 43 cases. Fifty-two tumors (72.2%) expressed one or more basal-like markers (CK5/6 and EGFR) (Figures 4a, b), and 43 (60.6%) expressed one or more myoepithelial markers (SMA, S-100, CD10, p63) (Figure 4c); however, 40 of these 43 also expressed basal-like markers. Basal-like and myoepithelial markers were detected in 26.7% and 16.7% of controls, respectively. Basal-like and myoepithelial markers were expressed significantly more frequently in patients with CNC compared with controls (P < 0.05). However, there was no significant association between the expression of basal-like and myoepithelial markers and the relative size of the necrotic zone (P > 0.05) (Table 2). Regarding molecular typing, 37 cases (50.7%) of CNC were basal-like subtype, while luminal A, luminal B, HER2-overexpressing, and null subtype accounted for 20 (27.4%), seven (9.6%), six (8.2%) and three cases (4%), respectively. There was no significant association between the molecular classification of CNC and the relative size of the necrotic zone (P > 0.05) (Table 2).

## Discussion

CNC has recently been described as a breast tumor subtype, but has not yet been recognized by WHO. In 1997, Hasebe et al. [1] first reported a series of patients with “fibrotic focus in invasive ductal carcinoma of the breast”. In 1999, Tsuda et al. [2] reported on a series with high-grade IDCs with large central acellular zones. They noted that these tumors carried high risks of metastasis and death. In 2001, Jimenez et al. [4] reported a similar type of breast cancer and named these tumors “centrally necrotizing carcinomas of the breast”. However, the largest of these reports only accounted for 34 cases. In the present study, we investigated 73 cases of CNC, which represents the largest series to date.

### Clinical manifestations

CNC accounts for 2%–3% of all breast cancers and occurs predominantly in middle-aged and older women [3]. The age of onset ranged from 30–80 years, with an average age of 51.8 years [3]. All patients in the current study were female, ranging in age from 34 to 80 years (mean age, 51.8 years), accounting for 2.7% of all breast carcinomas at our hospital during the study period, and consistent with previous reports. In our study, the clinical genetic testing or strong family history were not available, so BRCA1 mutation status was unknown for these cases and further research is needed. Jimenez et al. [4] reported that most cases were T1 or T2 tumors, and 72% were node-negative and only 28% had lymph node metastases, all fewer than four. In the current study, 64.5% (40/62) of patients were axillary node negative and 35.5% (22/62) had axillary lymph node metastases (mean, 5.8) which were in accordance with what was previously published. However, Tsuda et al. [3] found few lymph node-negative patients, and lymph node metastases were detected in 55% of patients, with a mean of 7.20. Reports on the radiographic features are scarce. In our study, ultrasonography was performed in 26 patients, usually presented with well-defined, heterogeneous, hypoechoic lesions. Oda et al. [7] reported a well-circumscribed tumor with cystic and solid parts and an abundant blood supply, similar to some in the present series, and noted that preoperative diagnosis was important for breast surgeons and radiologists because of the suspected risk of hemorrhage in these lesions. Hemanz et al. [5] reported one case of CNC in which it was not possible to obtain a sample of viable tumor cells despite numerous biopsies, because of the minimal width of the viable tumor rim and the predominance of fibrosis and adiponecrosis in the central region. In our study, two cases could not be diagnosed as breast carcinoma by needle core biopsy, but were subsequently diagnosed after complete tumor resection. We therefore recommend open biopsy in those cases suspicious for CNC with an inconclusive needle core biopsy, to avoid misdiagnosis.

### Pathological features

Yu et al. [6] reported well-circumscribed, nodular masses with a cut surface with a moderate or soft consistency and white to tan appearance, ranging in size from 1.0–5.0 cm (mean, 2.5 cm). In our study, 91.8% (59/73) of tumors were unicentric, well-defined nodules with pushing borders, ranging from 1.0–6.5 cm (mean, 2.49 cm). The cut surface was usually moderate to hard, gray, sallow, or reddish-brown, with necrosis or cystic degeneration in the central region, similar to the report of Yu et al. [6]. Infiltrative edge was only detected in 14 cases (19.2%) in our series, which was similar to that reported by Tsuda et al. [3].

Microscopically, the tumors demonstrated extensive central necrosis with varying levels of fibrotic or hyalinized matrix, surrounded by a ring of remaining tumor tissue. The transition between the central necrotic zone and the viable tumor tissue was usually abrupt, with no granulation tissue or collagen. These represented the most characteristic histological manifestations of CNC [4]. The central necrotic zone usually accounted for >70% of the tumor size, but only >30% in a few cases. The central necrotic or acellular zone showed three different morphological manifestations as described above, characterized by: (i) coagulative necrosis; (ii) fibrotic, hyaline material or scar tissue; and (iii) infarction. In our study, 63.4% of patients showed coagulative necrosis (47 cases), which was similar to the 68.6% reported by Yu et al. [6]. The morphological manifestations of the necrosis was also similar in both studies. The viable tumor tissue surrounding the central necrotic zone was poorly differentiated, being mostly IDC grade 3, with several cases of IDC grade 2 [6]. The periphery of the CNCs was usually associated with a variable ductal carcinoma in situ component [2]. Jimenez et al. [4] detected ductal carcinoma in situ in 19 cases (55.9%). In our series, IDC grade 3 was detected in 67 cases (91.8%), with ductal carcinoma in situ in 36 cases, similar to the findings of Jimenez et al. [4]. However, it is worth noting that some unusual histologic types of viable tumor tissue were also detected in our study, including invasive micropapillary carcinoma in two cases (first reported by Yu et al. [6]), intraductal papillary carcinoma in one case, and mucinous carcinoma in five cases (not previously described). Several cases have been accompanied by squamous metaplasia or spindle cell differentiation [3-5]. In this study, squamous metaplasia was present in four cases, spindle cell differentiation in one, and cartilaginous metaplasia in one (had not been published in the literature). However, differences in terms of the lymphocytic infiltrates and myxoid matrix which usually considered to be from myoepithelial cells of the breast [12,13] have been reported. Tsuda et al. [3] found that the central zone of most tumors was accompanied by a myxoid matrix, while only eight cases (24.2%) reported by Yu et al. displayed this feature [6], and 54.5% of cases were accompanied by lymphocytic infiltrates. However, peripheral lymphocytic infiltrates were rare in Jimenez et al.’s study [4]. In the present study, 38.4% of tumors displayed myxoid matrix and 72.6% showed peripheral lymphocytic infiltrates. Furthermore, 67.1% of cases were accompanied by granulomatous reactions at the periphery of the tumor, representing the first report of this feature.

CNC comprises an extensive central necrotic or acellular zone, but the definition of what proportion of the tumor area qualifies as “large” remains controversial. Tsuda et al. [3] considered that a central infarction occupying >30% was a large central acellular zone, while Jimenez et al. [4] suggested that the central necrotic or acellular zone should account for ≥70% of the cross-sectional area of the tumor. In the present study, the necrotic zone accounted for >30% of the cross-sectional area in all cases, and >70% in 41 cases (56.2%). However, there was no significant relationship between clinicopathological features and size of the necrotic zone (P > 0.05), suggesting that a central acellular zone accounting for >30% of the tumor provided an appropriate definition of CNC. However, other features should also be taken into consideration, including the characteristic histological manifestation of the extensive central necrotic or acellular zone surrounded by a ring of remaining tumor tissue. The pathogenesis of the central necrotic zone remains unclear. Jimenez et al. [4] suggested that rapid centrifugal growth with insufficient vascularization resulted in infarction. Proliferation of viable tumor cells (Ki-67 > 50% in 84.9% of CNCs and >70% in 58.8% in the present study), the myxoid matrix, and limitation and expansion of the surrounding granuloma may all contribute to the development of the central necrotic zone.

Although CNC was associated with a high rate of distant metastases, lymph node metastasis and vascular invasion were uncommon. Jimenez et al. [4] found that 72% of cases were node-negative, and vascular invasion only occurred in two cases (5.9%). Yu et al. [6] reported negative lymph node metastasis in 17 cases (58.6%) and vascular invasion in seven. In accordance with those studies, we found negative lymph nodes in 40 cases (64.5%) and vascular invasion in 10 (13.7%). However, in the present study, 67.1% of cases were accompanied by granulomatous reactions at the periphery of the tumor in which there were the regeneration of considerable capillary vessels and indicated that the newborn capillaries might supply nutrition for tumors as well as increased the risk of a blood metastasize.

### Immunophenotype

The immunophenotypic characteristics of CNC are unclear, including the expression patterns of ER, PR and HER2, and of basal-like and myoepithelial markers. The relationship between CNC and basal-like breast carcinoma is also uncertain. Tsuda et al. [3] reported that 85% of the tumors expressed myoepithelial markers (expression of ≥1 myoepithelial markers, including S100-α, S100-β, α-SMA and CK14), and speculated that the tumors might be derived from myoepithelial cells or undergo myoepithelial differentiation. Yu et al. [6] speculated that CNC might originate from basal-like cells and represent a basal-like breast carcinoma, because of the higher expression of basal-like markers (87.9%) and the fact that myoepithelial markers were often (46.2%) simultaneously positive for basal-like markers. Our study found higher expression of basal-like markers (72.2%) than myoepithelial markers (60.6%), but both were expressed more in CNCs than in controls. According to the molecular classification, more than half the CNCs (50.7%) were basal-like breast carcinomas, followed by luminal-A tumors (27.4%). These results were comparable to those of Yu et al. [6], supporting the idea that the tumors might be derived from basal-like cells or undergo basal-like differentiation. Although most CNCs were basal-like according to molecular-typing methods, further studies are needed to determine whether or not they should be included in basal-like breast cancer.

### Prognosis

CNC is characterized by aggressive behavior and a poor prognosis, with high rates of distant metastasis and mortality. Tsuda et al. [3] reported 20 cases of high-grade IDC with large, central acellular zones, compared with controls without these zones, and found that the rates of lung metastasis (65%) and brain metastasis (30%), as well as mortality, were higher than those in controls. Jimenez et al. [4] reported an OS of only 22.5 months in 34 patients with CNC, and of 20 patients with metastases, 15 developed lung and central nervous system metastases. In the present study, follow-up data were available for 28 patients. Disease progression occured in 11 patients, and by the last follow-up, one patient had died of breast carcinoma. The prognosis was poorer than in the 11 controls with follow-up data, who showed no local recurrence or metastasis. As well all know, necrosis and nodal status are ralated to the prognosis of breast cancer patients [14-17], but the independent risk factors for CNC remain controversial. Tsuda et al. [3] demonstrated that nodal status and a large, central acellular zone were independent prognostic factors for metastasis and death, and noted that the central acellular zone was a single indicator of a high risk of brain metastasis. Meanwhile, Jimenez et al. [4] noted that nodal status was not associated with survival but larger tumour size with poor prognosis. The present study showed that CNCs with necrotic zones accounting for >70% of the tumor had higher recurrence and metastasis rates than tumors in which the necrotic zone occupied >30% but <70% of the tumor, though the results were not significant. These results suggest that the size of the zone central necrotic or acellular zone should not be considered as an independent prognostic factor. However, the follow-up data were limited, and further studies involving more cases and longer follow-up times are needed to clarify the independent prognostic factors in CNC.

To summarize, CNC is characterized by the following features. (i) The tumors are usually well-defined, unicentric nodules with necrosis or cystic degeneration in the central region. (ii) Microscopically, the tumors comprise extensive central necrotic or acellular zones that account for ≥30% of the cross-sectional area of the tumor, surrounded by a rim of viable tumor cells, which are mostly high-grade IDCs. The transition between the central necrotic or acellular zone and viable tumor tissue is usually abrupt and the peripheral interstitium usually includes lymphocytic infiltrates and myxoid matrix. (iii) Proliferating granulation is usually present at the periphery of the tumor. (iv) Most tumor cells that demonstrate high proliferative activity express basal-like markers, and most tumors were basal-like breast cancer. (v) The age of onset was older and patients showed rapid clinical progression and a poor prognosis with high rates of recurrence and a tendency to develop lung and brain metastases.

## Conclusions

CNC represents a rare, novel subtype of breast carcinoma. It is associated with distinctive clinicopathologic features mostly characterized as basal-like type. Its high proliferative activity, highly-aggressive biological behavior, and high rates of recurrence and metastasis, suggest that CNC should be classified as a new type of breast carcinoma. Increasing awareness of this new type breast carcinoma will help to improve its diagnosis and therapy.

## Abbreviations

CNC: Centrally necrotizing carcinomas of the breast
IDC: Infiltrating ductal carcinoma
WHO: World Health Organization
ER: Estrogen receptor
PR: Progesterone receptor
EGFR: Epidermal growth factor receptor
CK: Cytokeratin
SMA: Smooth muscle actin
DFS: Disease-free survival
OS: Overall survival
BI-RADS: Breast imaging reporting and data system

## References

[1] Hasebe T, Tsuda H, Tsubono Y, Imoto S, Mukai K (1997). Fibrotic focus in invasive ductal carcinoma of the breast: a histopathological prognostic parameter for tumor recurrence and tumor death within three years after the initial operation. Jpn J Cancer Res.

[2] Tsuda H, Takarabe T, Hasegawa T, Murata T, Hirohashi S (1999). Myoepithelial differentiation in high—grade invasive ductal carcinomas with large central acellular zones. Hum Pathol.

[3] Tsuda H, Takarabe T, Hasegawa F, Fukutomi T, Hirohashi S (2000). Large,central acellular zones indicating myoepithelial tumor diferentiation in high—grade invasive ductal carcinomas as markers of predisposition to lung and brain metastases. Am J Surg Pathol.

[4] Jimenez RE, Wallis T, Visscher DW (2001). Centrally necrotizing carcinomas of the breast: a distinct histologic subtype with aggressive clinical behavior. Am J Surg Pathol.

[5] Hemanz F, Alonso-Bartolomé P, González-Rodilla I (2012). Centrally necrotizing breast carcinoma: a rare histological subtype, which was cause of misdiagnosis in an evident clinical local recurrence.World J Surg. Oncology.

[6] Yu L, Yang W, Cai X, Shi D, Fan Y, Lu H (2010). Centrally necrotizing carcinoma of the breast: clinicopathological analysis of 33 cases indicating its basal-like phenotype and poor prognosis. Histopathology.

[7] Oda K, Satake H, Nishio A, Ichihara S, Shimoyama Y, Imai T, Nagino M (2008). Radiologic–pathologic conferences of the Nagoya University Hospital: centrally necrotizing carcinoma of the breast. AJR Am J Roentqenol.

[8] Elston CW, Ellis IO (1991). Pathological prognostic factors in breast cancer. I. The value of histological grade in breast cancer:experience from a large study with long—term follow—up. Histopathology.

[9] Nielsen TO, HSU FD, Jensen K, Cheang M, Karaca G, Hu Z, Hernandez-Boussard T, Livasy C, Cowan D, Dressler L, Akslen LA, Ragaz J, Gown AM, Gilks CB, van de Rijn M, Perou CM (2004). Immunohistochemical and clinical characterization of the basal-1ike subtype of invasive breast carcinoma. Clin Cancer Res.

[10] Guideline Recommendations for HER2 Detection in Breast Cancer Group (2014). Guidelines for HER2 detection in breast cancer, the 2014 version. Zhonghua Bing Li Xue Za Zhi.

[11] Carey LA, Perou CM, Livasy CA, Dressler LG, Cowan D, Conway K, Karaca G, Troester MA, Tse CK, Edmiston S, Deming SL, Geradts J, Cheang MC, Nielsen TO, Moorman PG, Earp HS, Millikan RC (2006). Race, Breast cancer subtypes, and survival in the Carolina Breast Cancer Study. JAMA.

[12] Hamperl H (1970). The myoepithelia (myoepithelial cells): normal state; regressive changes; hyperplasia; tumors. Curr Top Pathol.

[13] Allen AC (1940). So-called mixed rumors of the mammary gland of dog and man, with special reference to the general problem of cartilage and bone formation. Arch Pathol.

[14] Gilchrist KW, Gray R, Fowble B, Tormey DC, Taylor SG (1993). Tumor necrosis is a prognostic predictor for early recurrence and death in lymph node positive breast cancer: a 10-year follow-up study of 728 Eastern Cooperative Oncology Group patients. J Clin Oncol.

[15] Georgescu R, Coroş MF, Stolnicu S, Podeanu D, Sorlea S, Roşca A, Copotoiu C (2012). Prognostic factors in breast cancer. Rev Med Chir Soc Med Nat Iasi.

[16] Xu Z, Marko NF, Angelov L, Barnett GH, Chao ST, Vogelbaum MA, Suh JH, Weil RJ (2012). Impact of preexisting tumor necrosis on the efficacy of stereotactic radiosurgery in the treatment of brain metastases in women with breast cancer. Cancer.

[17] Yenidunya S, Bayrak R, Haltas H (2011). Predictive value of pathological and immunohistochemical parameters for axillary lymph node metastasis in breast carcinoma. Diagn Pathol.

